# Supplementary material for: Association between oral microbiome and seven types of cancers in East Asian population: a two-sample Mendelian randomization analysis
Source: Front Mol Biosci. 2023 Nov 21;10:1327893. doi: 10.3389/fmolb.2023.1327893 (PMC10702768; doi:10.3389/fmolb.2023.1327893)
Supplement: Supplementary file 1 [file DataSheet1.ZIP › Supplementary Materials/MR plots for tongue/tongue═╝/Pancreatic cancer/╥╚╧┘░⌐_╔╕╤í╜ß╣√/pheno.2888_to_pancreatic cancer_forest.pdf]

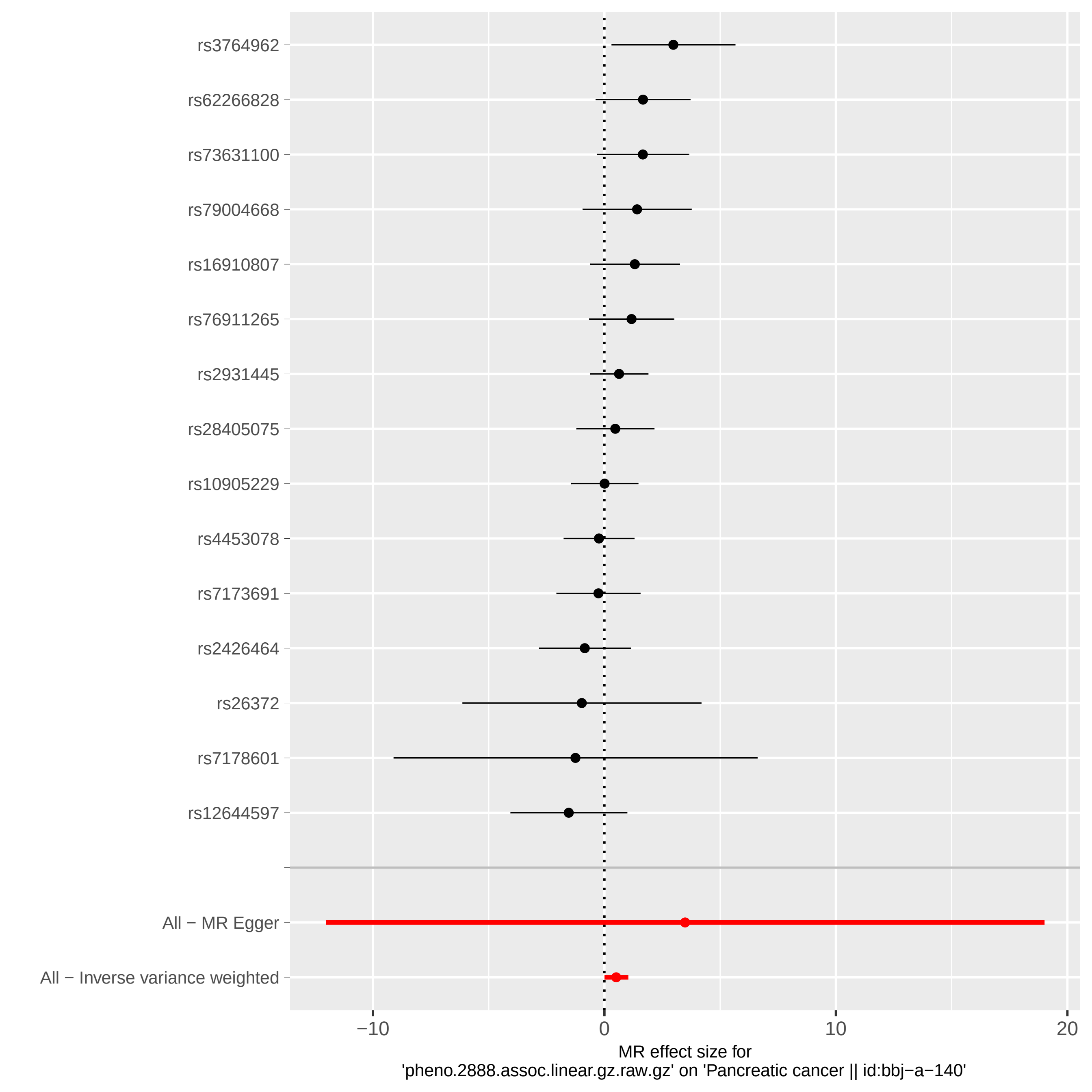
rs3764962
rs62266828
rs73631100
rs79004668
rs16910807
rs76911265
rs2931445
rs28405075
rs10905229
rs4453078
rs7173691
rs2426464
rs26372
rs7178601
rs12644597
All − MR Egger
All − Inverse variance weighted
−10
0
10
20
MR effect size for
'pheno.2888.assoc.linear.gz.raw.gz' on 'Pancreatic cancer || id:bbj−a−140'
